# The role of the α7 nicotinic acetylcholine receptor in promoting M2 macrophage polarization at inflammatory sites

**DOI:** 10.1038/s41598-026-35757-2

**Published:** 2026-01-14

**Authors:** Taiki Mihara, Hiroshi Tanabe, Yuma Nonoshita, Yuki Yamakawa, Tamaki Kurosawa, Masatoshi Hori

**Affiliations:** Department of Veterinary Pharmacology, Graduate School of Agriculture and Life Sciences, The University of Tokyo, Bunkyo-ku, 113-8657 Tokyo Japan

**Keywords:** Rodent, Macrophages, M1/M2 macrophage polarization, Inflammation, α7 nicotinic acetylcholine receptor, Inflammation, Monocytes and macrophages

## Abstract

The α7 nicotinic acetylcholine receptor (α7nAChR) on macrophages exerts anti-inflammatory effects by suppressing the JAK/STAT and NF-κB pathways. Although the role of α7nAChR in immunoregulatory mechanisms in “individual” macrophages is established, studies on α7nAChR in an “overall population” of macrophages, including M1/M2 polarity, remain limited. Therefore, we examined the role of α7nAChR in M1/M2 polarity in inflammation. We generated peritonitis mouse models via LPS treatment and sterile intestinal manipulation in wild-type and α7nAChR-deficient mice. M1/M2 macrophage polarization was measured using PCR and flow cytometry. THP-1 and human peripheral blood mononuclear cells (hPBMC)-derived monocytes were treated with the α7nAChR agonist PNU-282987 during differentiation into M1/M2 macrophages. α7nAChR deficiency upregulated mRNA expression of the M1 marker and downregulated the M2 marker in a peritoneal cell population. Flow cytometry analysis revealed that the proportion of M2 macrophages in the peritoneal cell population decreased in α7nAChR-deficient mice in both models. In splenectomized LPS-treated wild-type mice, the proportion of M2 macrophages in the peritoneal cell population was reduced compared to that in sham-operated LPS-treated mice. The M2 marker *CD206* and *IL10* were upregulated in PNU-282987-treated THP-1 and hPBMC-derived macrophages. These results revealed that α7nAChR exerted M2-enhancing effects with the mechanism suggestively acting in the spleen.

**Supplementary Information:**

The online version contains supplementary material available at 10.1038/s41598-026-35757-2.

## Introduction

Inflammation is an essential biological response that eliminates pathogens and damaged tissues and is triggered by diverse factors, including infection and physical trauma. However, excessive inflammation can cause secondary tissue damage; thus, inflammation must be quickly regulated after the elimination of pathogens. Immune cells play a critical role in tightly controlling the inflammatory response, and macrophages are important owing to their involvement in both the initiation and resolution of inflammation by producing cytokines and growth factors^1,2^. Macrophages secrete cytokines and chemokines to induce acute inflammation in the early inflammatory phase, whereas they produce anti-inflammatory cytokines and growth factors to ameliorate inflammation and promote tissue repair during the recovery phase^3^. This unique property is attributed to their ability to exhibit two highly plastic phenotypes: M1 macrophages, which produce pro-inflammatory cytokines, and M2 macrophages, which exhibit anti-inflammatory and tissue repair properties^3^. The balance between M1 and M2 macrophages is referred to as M1/M2 polarity. M1 macrophages dominate the early phase of inflammation, whereas M2 macrophages become prevalent during the recovery phase^4^.

Acetylcholine receptors were initially discovered as the neuronal receptors responsible for neurotransmission. They consist of five subunits, the subtypes of which vary depending on the combination of the subunits^5^. The α7 nicotinic acetylcholine receptor (α7nAChR) is a homodimer comprising five α7 subunits and is abundantly expressed in the hippocampus, where it is involved in learning and memory^6^. In 2003, it was reported that α7nAChR is also expressed on macrophages, and its activation can exert anti-inflammatory effects and ameliorate septic shock in mice^7^. These findings led to the discovery of the “cholinergic anti-inflammatory pathway,” which is mediated by the activation of α7nAChR on macrophages following parasympathetic stimulation^7,8^. Specifically, noradrenaline released from the posterior terminal of the vagus nerve into the spleen during vagal excitation promotes acetylcholine production in CD4-positive T cells. This subsequently exerts anti-inflammatory effects by activating α7nAChR on macrophages and inhibiting the release of inflammatory cytokines by suppressing the JAK/STAT and NF-κB pathways^8,9^. Although the role of α7nAChR in immunoregulatory mechanisms in “individual” macrophages has been demonstrated, studies on α7nAChR in an “overall population” of macrophages, including M1/M2 polarity, remain limited. Therefore, we elucidated the potential role of α7nAChR in M1/M2 polarity regulation in infectious and non-infectious inflammation.

## Materials and methods

### Animals

Male and female C57BL/6JJmsSlc mice (wild-type) obtained from Sankyo Labo Service (Hamamatsu, Japan), and B6.129S7-*Chrna7*^*tm1Bay*^/J mice (α7nAChR-deficient mice) from the Jackson Laboratory (Bar Harboe, Maine, USA), aged 7–9 weeks, were used in this study. Animals were randomly allocated into each group by arbitrarily selecting mice from the same housing cages, in which they were maintained under identical conditions, without considering the order or physical conditions. Data collection and analysis were performed under blind conditions, except for surgical interventions such as splenectomy, where blinding was not feasible due to the nature of the procedure. The mice were bred under controlled conditions at a temperature of 25 °C and a 12-h light/dark cycle with free access to feed (MF; Oriental Yeast Co., Tokyo, Japan) and water. The diet composition is described in Supplementary Table S1. We used Q-Pla Tip (Sankyo Labo Service) for bedding and TAR-100E-A (Toyo-riko, Tokyo, Japan) for the caging system. Three or four mice were co-housed in each cage for a one-week familiarization period before being used in the experiment. Mice were not fasted before conducting challenges or assessments. All interventions were performed during the light cycle (8:00–20:00 h). All experimental procedures for animal handling were conducted in accordance with the Guide for Animal Use and Care published by the University of Tokyo and the International Animal Research: Reporting of In Vivo Experiments (ARRIVE) guidelines, and were approved by the Institutional Review Board of the University of Tokyo (approval code: P22-091H02). Permission to use α7nAChR-deficient mice was obtained from the Institutional Review Board of the University of Tokyo (approval code: L22-028).

### Peritonitis mouse model

LPS-induced peritonitis mouse models of infectious inflammation and intestinal manipulation (IM) peritonitis mouse models of non-infectious inflammation were generated to elucidate the role of α7nAChR in M1/M2 polarity. To generate the LPS peritonitis model, mice were intraperitoneally administered 1 mg/kg of LPS (L2880; Sigma-Aldrich, St. Louis, MO, USA) or physiological saline as vehicle. LPS and vehicle were administered at a volume of 10 ml/kg using a 29-gauge needle (SS-10M2913A; Terumo, Tokyo, Japan) and peritoneal cell populations were collected at 3, 24, 48, and 72 h after injection. To generate the IM peritonitis model, we manipulated the intestinal tract under anesthesia as described previously^10^. Briefly, the distal ileum was exteriorized and scrubbed using a sterile cotton swab moistened with physiological saline under anesthesia with a three-agent mixed anesthetic, comprising medetomidine (0.75 mg/kg; Dorbene vet, Kyoritsu Seiyaku, Tokyo, Japan), midazolam (4 mg/kg; NIG, Takeda Yakuhin, Osaka, Japan), and butorphanol (5 mg/kg; Vetorphale, Meiji, Tokyo, Japan)). After surgery, anesthesia was antagonized by atipamezole (Antisedan, Nippon Zenyaku, Fukushima, Japan) administration. In this model, it has been reported that peritoneal macrophages become activated and produce various inflammatory cytokines within the peritoneal cavity, which represents a key pathological event in peritonitis^11^. Peritoneal cell populations were collected at 3, 24, and 48 h after manipulation using a cotton swab.

### Splenectomy

The abdominal walls of the mice were opened under isoflurane anesthesia. The splenic arteries and veins were ligated using 5 − 0 silk thread at the splenic hilum, and the spleen was subsequently removed. Sham-operated mice underwent laparotomy without splenectomy on the same day. Three weeks following the splenectomy, both the splenectomized and sham-operated mice underwent LPS treatment.

### Isolation of peritoneal cell population

Following euthanasia under deep isoflurane anesthesia, the peritoneal cavity was flushed with 5 mL of DMEM (041-29775; Wako, Osaka, Japan). Subsequently, the obtained lavage fluid was centrifuged (700 × *g*, 5 min), and the resulting pellet was washed with RBC lysis buffer (420301; BioLegend, San Diego, CA, USA). After centrifugation, the obtained cells were used as the peritoneal cell population for the subsequent experiments.

### Cell culture

The human monocytic leukemia cell line THP-1 was obtained from the RIKEN BioResource Research Center (RCB3686; Ibaraki, Japan). THP-1 cells were cultured as described previously with a few modifications^12^. Briefly, THP-1 cells were seeded in 6-well plates (3.0 × 10^5^ cells/well) and cultured in RPMI-1640 (189–02025; Wako) containing 10% FBS (10270106, Lot.: 42Q3082K; Thermo Fisher Scientific, Waltham, MA, USA), 100 U penicillin, and 100 µg/mL streptomycin (15140148; Thermo Fisher Scientific). Differentiation of monocytes into macrophages was induced using 20 ng/mL PMA (Phorbol 12-myristate 13-acetate, 162-23591; Wako) for 24 h. M1 and M2 macrophage differentiation was induced by incubating cells with 100 ng/mL IFN-γ (093-05631; Wako) and 40 ng/mL IL-4 (098-03964; Wako), respectively, for 48 h.

Human peripheral blood mononuclear cells (hPBMC) were purchased from PRECISION (10 million/vial, Subject ID: 2504475, Lot: 3010116155). The cells were seeded at a density of 1.6 × 10^6^ cells per well in 12-well plates and incubated for 1.5 h in monocyte attachment buffer (C-28051; Takara). After attachment, the cells were cultured in RPMI-1640 supplemented with L-glutamine, HEPES (189–02145, Wako), 10% FBS (10270106, Lot: 42Q3082K; Thermo Fisher Scientific), 100 U penicillin, and 100 µg/mL streptomycin (15140148; Thermo Fisher Scientific) for 6 days, with medium changes every 2 days. Monocyte differentiation into macrophages was induced by adding 100 ng/mL GM-CSF (139-19431; Wako). For M1 and M2 differentiation, cells were incubated with 100 ng/mL IFN-γ (093-05631; Wako) or 40 ng/mL IL-4 (098-03964; Wako), respectively, for 48 h.

The α7nAChR agonist PNU-282987 (10 µM, ab120558; Abcam, Cambridge, UK) was administered simultaneously with M1 or M2 differentiation in both THP-1-derived and hPBMC-derived macrophages. CD68, CD86, and CD206 were used as macrophage, M1, and M2 markers, respectively^13^.

### mRNA expression measurement

Peritoneal cell populations were collected from mice 3, 24, 48, and 72 h after inflammation model generation. Total RNA was extracted using TRI Reagent (TR118: Molecular Research Center, Cincinnati, OH, USA) according to the manufacturer’s instructions. Total RNA was reverse-transcribed using ReverTra Ace (TRT-101; TOYOBO, Shiga, Japan) and random 9-mer primers (FSK-301; TOYOBO) at 30 °C for 10 min, 42 °C for 1 h, and 99 °C for 5 min. Real-time PCR was performed on an Aria Mx Real-Time PCR System (Agilent, Santa Clara, CA, USA) using THUNDERBIRD Next SYBR Green (QPX-201; TOYOBO) under the following conditions: denaturation at 95 °C for 1 min, 40 cycles of amplification at 95 °C for 15 s, and extension at 60 °C for 1 min. The relative expression levels of target genes were normalized to that of 18 S rRNA and calculated using the ΔΔCt method^14^. The primer sets and the expected sizes of the real-time PCR products are listed in Supplementary Table S2.

### Flow cytometry

The peritoneal cell population was suspended in PBS with 2% FBS at a cell concentration of 1.0 × 10^5^ cells per 100 µL. Propidium iodide (00-6990-50; Thermo Fisher Scientific) was used to remove the dead cells. To determine the proportions of M1 and M2 macrophages, macrophages were detected using the Alexa Fluor 488 CD68 antibody (0.5 µg per 1.0 × 10^5^ cells, FA-11, 53-0681-82; Thermo Fisher Scientific), M1 macrophages with the PE CD80 antibody (0.2 µg per 1.0 × 10^5^ cells, B7-1, 12-0801-81; Thermo Fisher Scientific), and M2 macrophages with the PE-Cy7 CD206 antibody (0.2 µg per 1.0 × 10^5^ cells, MR6F3, 25-2061-80; Thermo Fisher Scientific). To determine the proportion of dendritic cells, leukocyte populations were detected using the PE-Cy7 CD45 antibody (0.2 µg per 1.0 × 10^5^ cells, 30-F11, 406-0451-82; Thermo Fisher Scientific) and within this population, dendritic cells were identified using the FITC CD11c antibody (0.5 µg per 1.0 × 10^5^ cells, N418, FITC-65130; Thermo Fisher Scientific). After reacting with antibodies and propidium iodide at room temperature (25–28 °C) for 1 h in the shade, the samples were washed twice and analyzed using FACSVerse (BD Biosciences, Franklin Lakes, NJ, USA). The gating of each antibody was established using single staining samples and a negative control; for CD206 only, the higher value in the bimodal distribution was defined as M2 macrophages.

### Enzyme-linked immunosorbent assay (ELISA)

ELISA assays for acetylcholine, IL-4, and IFN-γ were performed using the Mouse Acetylcholine ELISA Kit (ab287812; Abcam), the Mouse IL-4 ELISA Kit (KE10010; Proteintech, Rosemont, IL, USA), and the Mouse IFN-γ ELISA Kit (KE10094; Proteintech), respectively, in strict accordance with the manufacturers’ instructions.

### Statistical analyses

Significant differences between groups were determined using one-way ANOVA, followed by Sidak multiple mean comparisons test (three groups) and Student’s *t*-test for comparisons between two groups. All analyses were performed using GraphPad Prism 9 (GraphPad Software, La Jolla, CA, USA), and data were considered significant at *p* ˂ 0.05.

## Results

### LPS and IM-treated α7nAChR-deficient mice showed increased expression of M1 markers and decreased expression of M2 markers in the whole peritoneal cell population

**Fig. 1:**
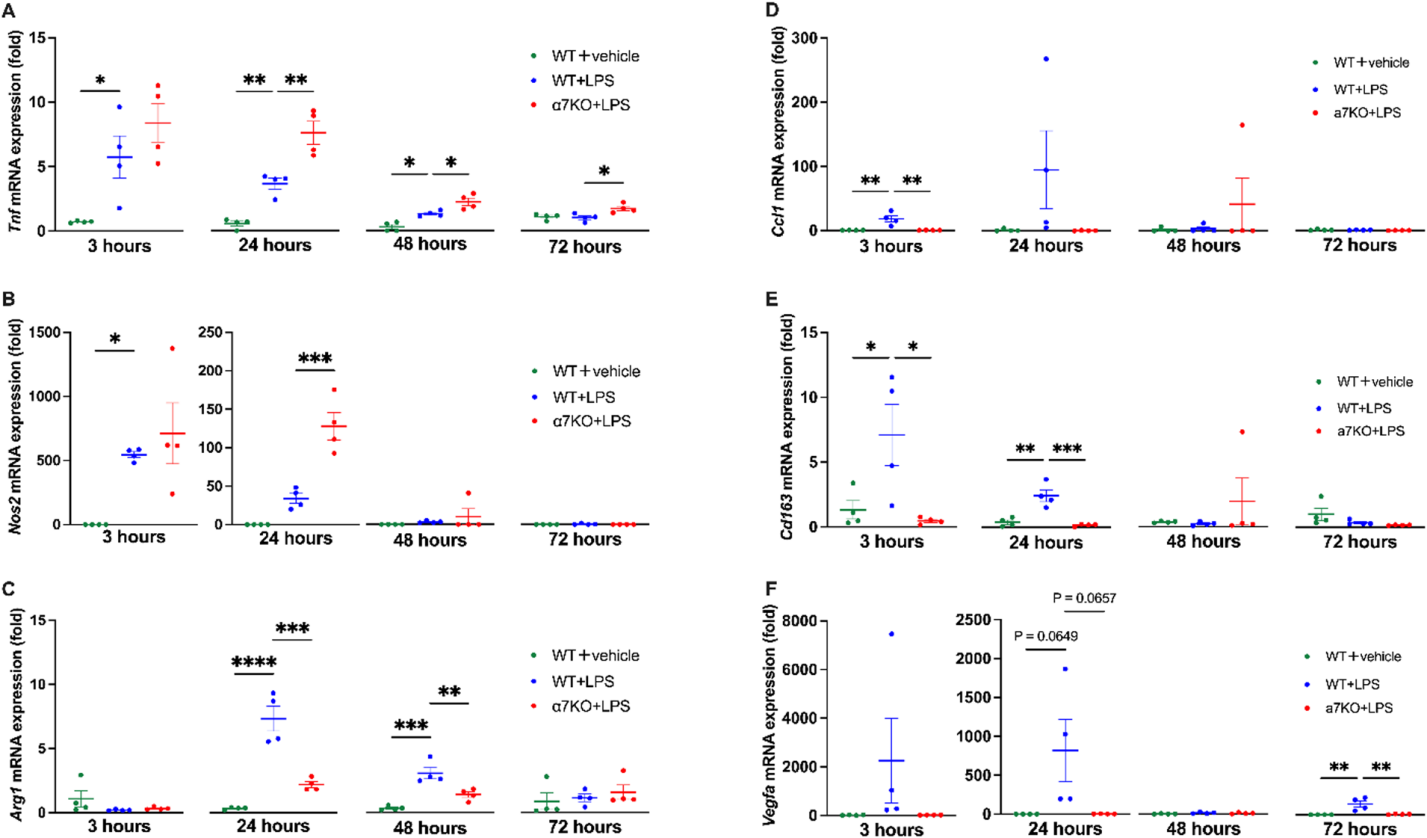
M1 and M2 marker expression in the peritoneal cell population derived from a mouse model of lipopolysaccharide (LPS)-induced peritonitis at different time points. mRNA expression of M1 macrophage markers, including (**A**) *Tnf* and (**B**) *Nos2*, and the M2a-d macrophage marker (**C–F**) *Arg1*, *Ccl1*,* CD163* and *Vegfa* at 3, 24, 48, and 72 h after LPS (1 mg/kg) i.p. administration (*N* = 4, respectively). *p < 0.05, **p < 0.01, ***p < 0.001, and ****p < 0.0001 denote significant differences. *Nos2*, nitric oxide synthase 2; *Arg1*, arginase; *Ccl1*, C-C motif chemokine ligand 1; *Cd163*, cluster of differentiation 163; *Vegfa*, vascular endothelial growth factor A; WT, wild-type; α7KO, α7 nicotinic acetylcholine receptor knockout.

To investigate the M1/M2 macrophage polarity during infectious inflammation, we established an LPS peritonitis model and measured the mRNA expression levels of the M1 macrophage markers *Tnf* and nitric oxide synthase 2 (*Nos2*) along with the M2a macrophage marker arginase-1 (*Arg1*), M2b marker C-C motif chemokine ligand 1 (*Ccl1*), M2c marker *CD163*, and M2d vascular endothelial growth factor A (*Vegfa*) in the whole peritoneal cell population. *Tnf* and *Nos2* mRNA expression levels peaked at 3 h, and *Arg1* expression levels peaked at 24 h after LPS administration (Fig. 1A-C, blue plots), followed by a gradual decrease. *Ccl1*, *Cd163*, and *Vegf* mRNA expression peaked between 3 and 24 h, then gradually declined toward 72 h (Fig. 1D-F, blue plots). These results showed that M1 polarity was dominant at 3 h after LPS treatment, whereas M2 polarity was dominant after 24 h.

To examine whether α7nAChR is involved in regulating M1/M2 polarity, we generated an LPS peritonitis model using α7nAChR-deficient mice. At 24 h after LPS administration, there was a significant increase in *Tnf* and *Nos2* mRNA expression and a significant decrease in *Arg1* mRNA expression in α7nAChR-deficient mice compared with that in wild-type mice (Fig. 1AC). Additionally, *Arg1* mRNA expression remained almost unchanged from 3 to 72 h in α7nAChR-deficient mice (Fig. 1C, red plots). Furthermore, at 3 h, *Ccl1* and *Cd163* expression levels were significantly decreased in α7nAChR-deficient compared with WT mice, and *Cd163* displayed a similarly marked reduction at 24 h (Fig. 1D, E). Although *Vegfa* expression exhibited large variability among individual mice, it was generally lower in α7nAChR-deficient than in WT mice (Fig. 1F). Additionally, in α7nAChR-deficient mice, *Ccl1*, *Cd163*, and *Vegfa* mRNA expression dynamics remained relatively unchanged from 3 h to 72 h, resembling the *Arg1* pattern (Fig. 1DF).

**Fig. 2:**
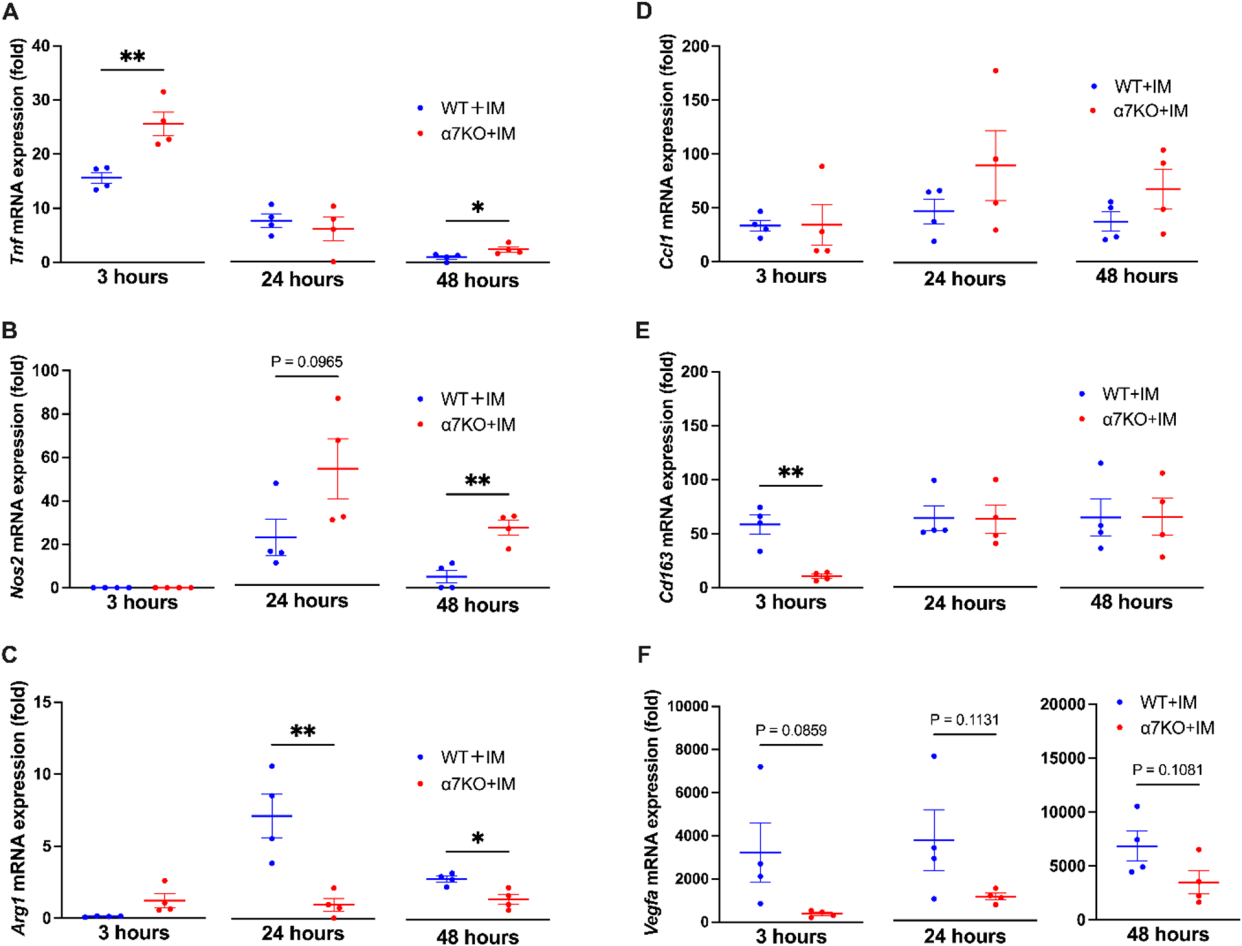
M1 and M2 marker expression in the peritoneal cell population derived from a mouse model of intestinal manipulation (IM) peritonitis at different time points. mRNA expression of M1 macrophage markers, including (**A**) *Tnf* and (**B**) *Nos2*, and the M2a-d macrophage marker (**C–F**) *Arg1*, *Ccl1*,* CD163* and *Vegfa* at 3, 24, and 48 h after IM (*N* = 4, respectively). *p < 0.05 and **p < 0.01 denote significant differences. IM, intestinal manipulation; *Nos2*, nitric oxide synthase 2; *Arg1*, arginase; *Ccl1*, C-C motif chemokine ligand 1; *Cd163*, cluster of differentiation 163; *Vegfa*, vascular endothelial growth factor A; WT, wild-type; α7KO, α7 nicotinic acetylcholine receptor knockout.

To investigate M1/M2 macrophage polarity during non-infectious inflammation, we established an IM peritonitis model. A previous study showed that inflammation in the IM model converges after 48 h^15^; therefore, we sampled the whole peritoneal cell population at 3, 24, and 48 h after establishing the model. *Tnf* and *Nos2* expression levels peaked at 3 and 24 h, whereas *Arg1* expression peaked at 24 h during non-infectious inflammation (Fig. 2A–C, blue plots) and the *Ccl1*, *Cd163*, and *Vegfa* mRNA expression exhibited high expression levels from 3 h to 48 h (Fig. 2DF). Notably, *Nos2* expression was completely abolished at 3 h during non-infectious inflammation, whereas *Nos2* was significantly upregulated at the same time point during infectious inflammation (Figs. 1B and 2B).

In α7nAChR-deficient mice, *Tnf* expression levels were significantly higher at 3 h, *Nos2* expression was significantly higher at 48 h (Fig. 2A, B), and *Arg1* expression was significantly lower at 24 and 48 h (Fig. 2C) than in wild-type mice. Consistent with the LPS peritonitis model, *Arg1* expression remained nearly unchanged in the IM peritonitis model (Fig. 2C, red plots). No significant differences were observed between WT and α7nAChR-deficient mice for *Ccl1* from 3 h to 48 h (Fig. 2D). In contrast, *Cd163* expression was significantly higher in WT mice than in α7nAChR-deficient mice at 3 h (Fig. 2E). Although *Vegfa* did not exhibit statistically significant differences, its expression consistently tended to be higher in WT than in α7nAChR-deficient mice from 3 h to 48 h (Fig. 2F).

Overall, these results suggest that α7nAChR was involved in regulating M2 polarity during infectious and non-infectious inflammation.

### M1/M2 Polarity of peritoneal macrophages showed M1 dominance in α7nAChR-deficient mice

Changes in the expression of the M1 and M2 macrophage markers in whole peritoneal cell populations could have been obscured by the presence of non-macrophage immune cells. Therefore, we used flow cytometry to specifically evaluate the M1/M2 polarization of peritoneal macrophages in LPS and IM-induced peritonitis models and provide a more detailed analysis.

First, wild-type and α7nAChR-deficient mice were injected intraperitoneally with saline, and the proportions of macrophages (CD68-positive) in the peritoneal cell population, M1 (CD80-positive) and M2 (CD206-positive) macrophages among the total macrophage population, and the M2/M1 ratio were measured at 24 and 48 h post-injection. No significant differences were found across all metrics between the two groups at both time points, indicating that saline administration did not influence macrophage polarization in the M1/M2 phenotype (Supplementary Fig. S1).

**Fig. 3:**
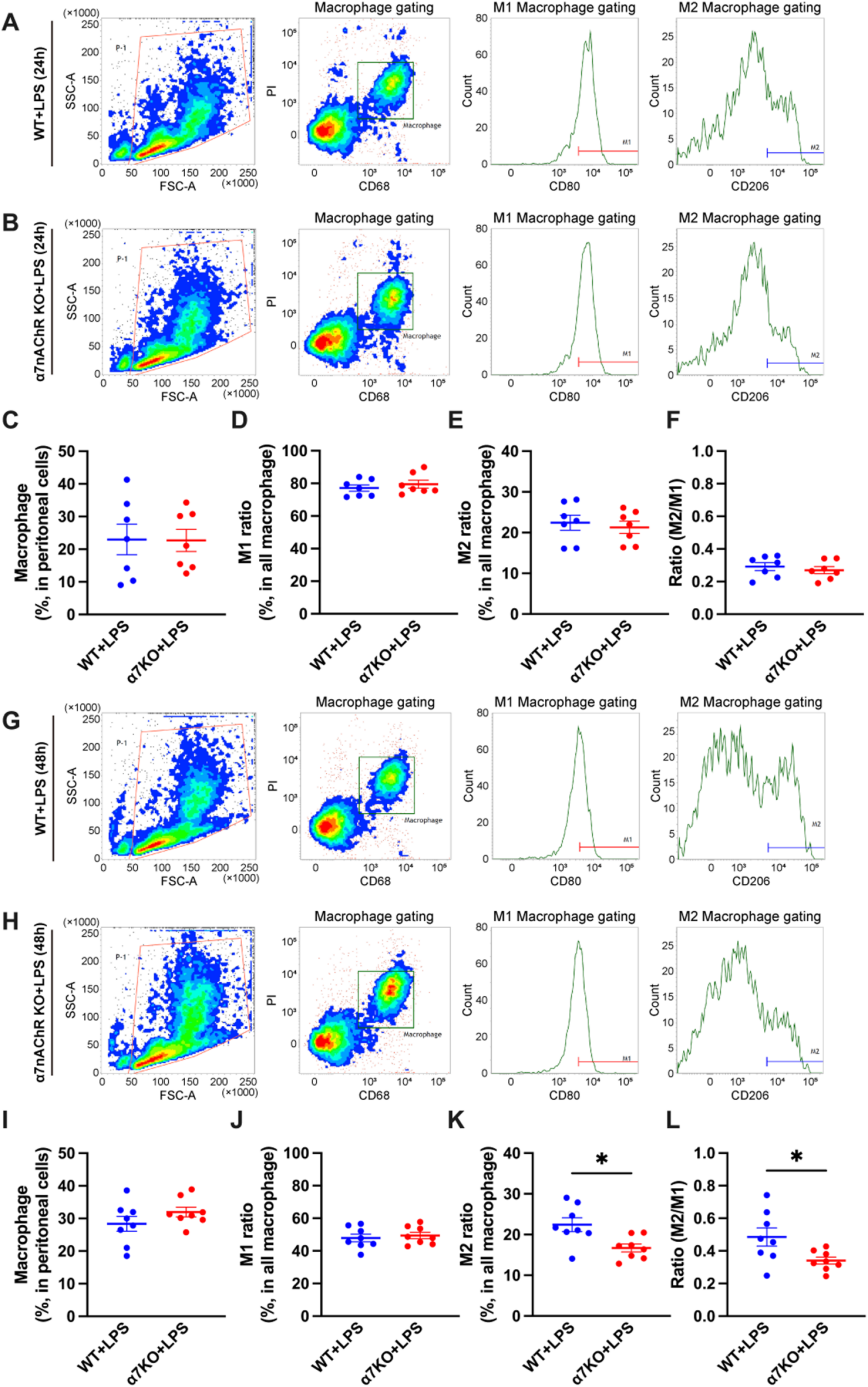
Proportion of M1 and M2 macrophages to total peritoneal macrophages and the M2/M1 ratio of LPS-induced peritonitis model mice generated from wild-type and α7nAChR-deficient mice. (**A**,** B**) FACS plots of peritoneal cell populations derived from WT and α7KO mice after 24 h of LPS treatment (1 mg/kg, i.p.). Proportions of (**C**) macrophages to the whole peritoneal cell population, (**D**) M1 macrophages to all macrophages, and (**E**) M2 macrophages to all macrophages (*N* = 7, respectively). (**F**) The M2/M1 ratio derived in (**D**) and (**E**) (*N* = 7). (**G**,** H**) FACS plots of peritoneal cell populations derived from WT and α7KO mice after 48 h of LPS treatment (1 mg/kg, i.p.). Proportions of (**I**) macrophages to the whole peritoneal cell population, (**J**) M1 macrophages to all macrophages, and (**K**) M2 macrophages to all macrophages (*N* = 8, respectively). (**L**) The M2/M1 ratio derived in (**J**) and (**K**) (*N* = 8). *p < 0.05 denotes significant differences. PI, Propidium iodide; WT, wild-type; α7KO, α7 nicotinic acetylcholine receptor knockout.

In the LPS-induced peritonitis models of both wild-type and α7nAChR-deficient mice, macrophages constituted approximately 20% of the total peritoneal cell population at 24 h post-LPS administration (Fig. 3A–C). The proportions of M1 and M2 macrophages among the total macrophage population did not differ between wild-type and α7nAChR-deficient mice, resulting in no change in the M2/M1 ratio (Fig. 3D–F). At 48 h post-LPS administration, macrophages accounted for approximately 30% of the peritoneal cell population in both groups (Fig. 3G–I). Although the proportion of M1 macrophages remained similar, α7nAChR-deficient mice exhibited a significant reduction in the proportion of M2 macrophages, leading to a corresponding significant decrease in the M2/M1 ratio (Fig. 3J–L).

**Fig. 4:**
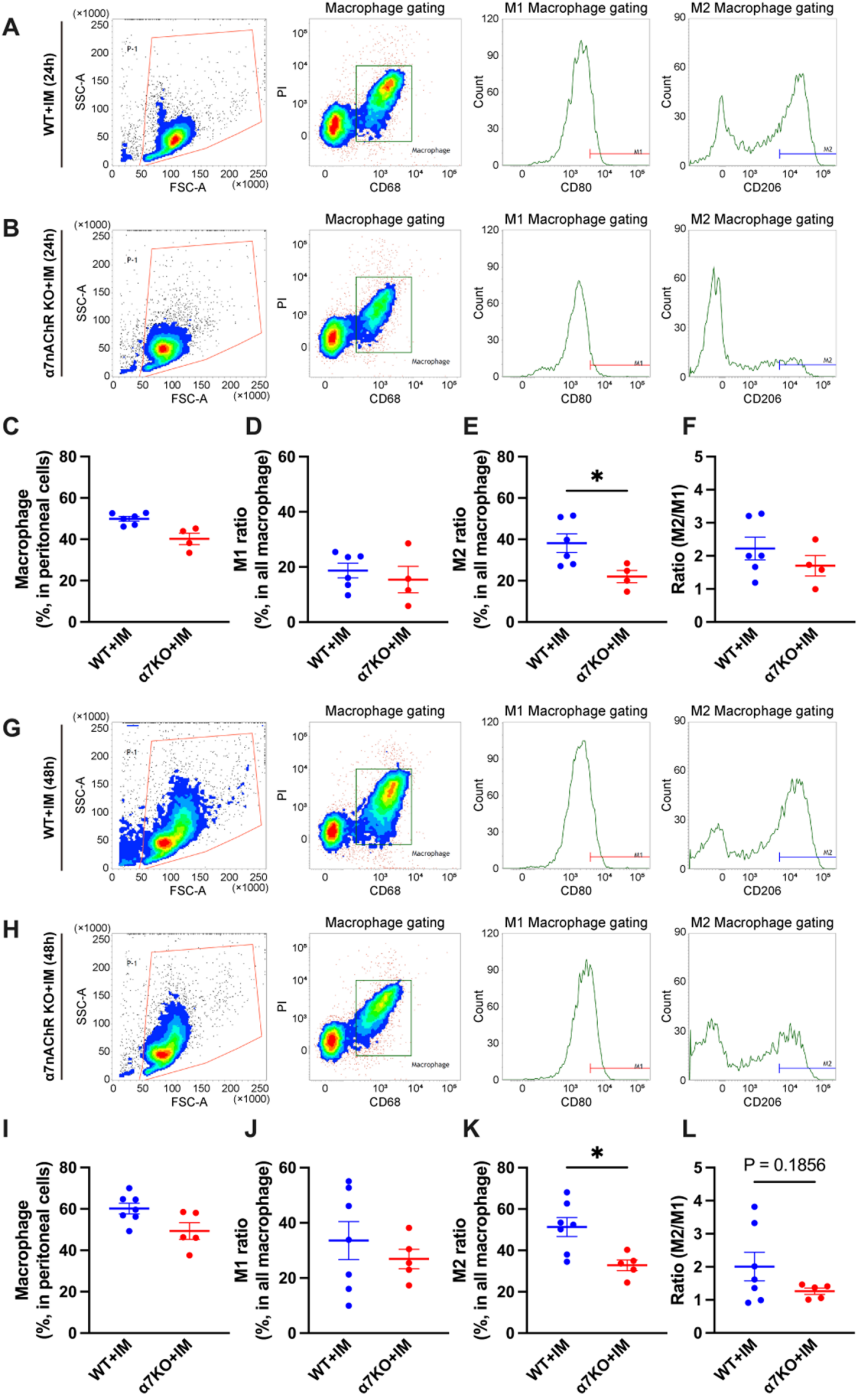
Proportions of M1 and M2 macrophages to total peritoneal macrophages and the M2/M1 ratio of IM-induced peritonitis model mice generated from wild-type and α7nAChR-deficient mice. (**A**,** B**) FACS plots of peritoneal cell populations derived from WT and α7KO mice 24 h after IM. Proportions of (**C**) macrophages to the whole peritoneal cell population, (**D**) M1 macrophages to all macrophages, and (**E**) M2 macrophages to all macrophages (*N* = 4–6, respectively). (**F**) The M2/M1 ratio derived in (**D**) and (**E**) (*N* = 4–6). (**G**,** H**) FACS plots of peritoneal cell populations derived from WT and α7KO mice 48 h after IM. Proportions of (**I**) macrophages to the whole peritoneal cell population, (**J**) M1 macrophages to all macrophages, and (**K**) M2 macrophages to all macrophages (*N* = 5–7, respectively). (**L**) The M2/M1 ratio derived in (**J**) and (**K**) (*N* = 5–7). *p < 0.05 denotes significant differences. IM, intestinal manipulation; PI, Propidium iodide; WT, wild-type; α7KO, α7 nicotinic acetylcholine receptor knockout.

In the IM-induced peritonitis models of both wild-type and α7nAChR-deficient mice, macrophages constituted approximately 50% of the total peritoneal cell population in both groups at 24 h after IM (Fig. 4A–C). The proportion of M1 macrophages did not differ between wild-type and α7nAChR-deficient mice (Fig. 4D). However, the proportion of M2 macrophages relative to the total macrophage population was significantly reduced in α7nAChR-deficient mice at 24 h, and the M2/M1 ratio was slightly reduced in α7nAChR-deficient mice compared to that in wild-type mice (Fig. 4E and F). At 48 h after IM, although the proportion of all macrophages to the whole peritoneal cell population and M1 macrophage proportion remained unchanged in both groups (Fig. 4G–J), the proportion of M2 macrophages remained significantly decreased in the α7nAChR-deficient group, and the M2/M1 ratio showed a similar trend toward reduction (Fig. 4K, L). These results indicated that α7nAChR was involved in the enhancement of M2 polarity.

### M2 macrophage Polarity at inflammation sites was enhanced in the spleen

Although certain M2 macrophages originate from tissue-resident macrophages, most M1 and M2 macrophages differentiate from monocytes under the influence of the local inflammatory environment^16,17^. Since α7nAChR is activated by acetylcholine, we hypothesized that acetylcholine levels would increase in the peritoneal cavity during inflammation, thereby promoting M2 macrophage differentiation by acting on recruited monocytes, which have been previously demonstrated to express α7nAChR^18,19^. To test this hypothesis, we quantified acetylcholine levels in peritoneal lavage fluid derived from LPS- and IM-induced peritonitis model mice; however, acetylcholine was undetectable in all samples (Supplementary Fig. S2A, B). Similarly, culture medium derived from 48 h-incubated peritoneal cell populations yielded the same results (Supplementary Fig. S2B). These findings suggest that acetylcholine levels are unlikely to increase within the peritoneal cavity, and therefore, may not activate α7nAChR to influence macrophage polarization.

Furthermore, we measured the proportions of dendritic cells, important for M1 and M2 macrophage differentiation, within peritoneal cell populations obtained from LPS- and IM-induced peritonitis models (Supplementary Fig. S3A). In both groups, dendritic cell proportions significantly increased in α7nAChR-deficient mice (Supplementary Fig. S3B–D), raising the possibility of altered IFN-γ and IL-4 levels at the inflammatory sites and of corresponding changes in M2 macrophage proportions. To test this hypothesis, we quantified IFN-γ and IL-4 concentrations in peritoneal lavage fluids from LPS- and IM-induced peritonitis models. However, we observed no significant changes (Supplementary Fig. S4A, B). Furthermore, to directly assess the cytokine-producing capacity of whole peritoneal immune cells, we measured IFN-γ and IL-4 concentrations in the culture medium of peritoneal cell populations isolated from LPS- and IM-induced peritonitis model mice using WT and α7nAChR-deficient mice, and the results similarly showed no significant changes (Supplementary Fig. S4C, D). These results suggest that the reduced M2 polarization observed in α7nAChR-deficient mice is unlikely to be mediated by changes in the proportion of dendritic cells or the peritoneal cytokine micro-environment.

**Fig. 5:**
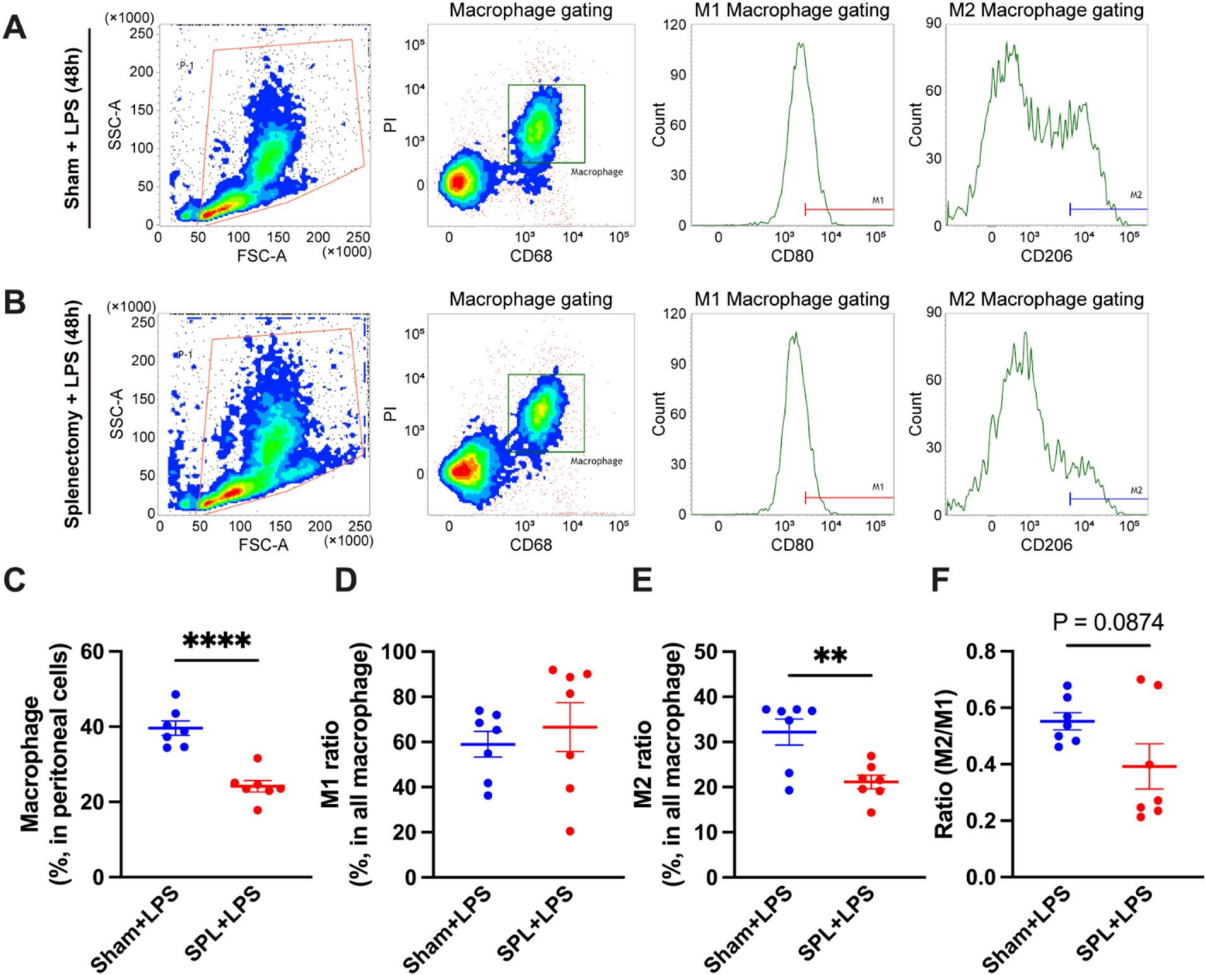
Proportion of M1 and M2 macrophages to total peritoneal macrophages and the M2/M1 ratio of LPS-induced peritonitis model mice generated from splenectomized wild-type mice. (**A**,** B**) FACS plots of peritoneal cell populations derived from sham-operated and splenectomized wild-type mice after 24 h of LPS treatment (1 mg/kg, i.p.). Proportions of (**C**) macrophages to the whole peritoneal cell population, (**D**) M1 macrophages to all macrophages, and (**E**) M2 macrophages to all macrophages (*N* = 6–7, respectively). (**F**) The M2/M1 ratio derived in (**D**) and (**E**) (*N* = 6–7). **p < 0.01 and ****p < 0.0001 denote significant differences. PI, Propidium iodide. SPL, Splenectomy.

As demonstrated in the anti-inflammatory reflex pathway, immune cells predominantly respond to acetylcholine in the spleen^7,8^. Therefore, we hypothesized that monocytes may undergo α7nAChR activation by acetylcholine in the spleen, acquire enhanced M2 differentiation potential, and subsequently be recruited to inflammatory sites with this pro-M2 property. To test this hypothesis, we developed a splenectomized mouse model of LPS-induced peritonitis and assessed the M1/M2 macrophage polarity in the peritoneal cavity using flow cytometry (Fig. 5A, B). The proportion of total macrophages to the whole peritoneal cell population was significantly decreased in splenectomized mice treated with LPS compared with that in sham-operated mice (Fig. 5C). Although the proportion of M1 macrophages to the total macrophage population remained unchanged after splenectomy (Fig. 5D), that of M2 macrophages was significantly reduced (Fig. 5E). This resulted in a shift in M1/M2 polarity toward M1 dominance (Fig. 5F). These findings suggest that the α7nAChR-mediated enhancement of M2 macrophage migration and polarization likely occurs in the spleen.

### Activation of α7nAChR enhanced M2 macrophage differentiation of THP-1 cells and hPBMC in vitro

**Fig. 6:**
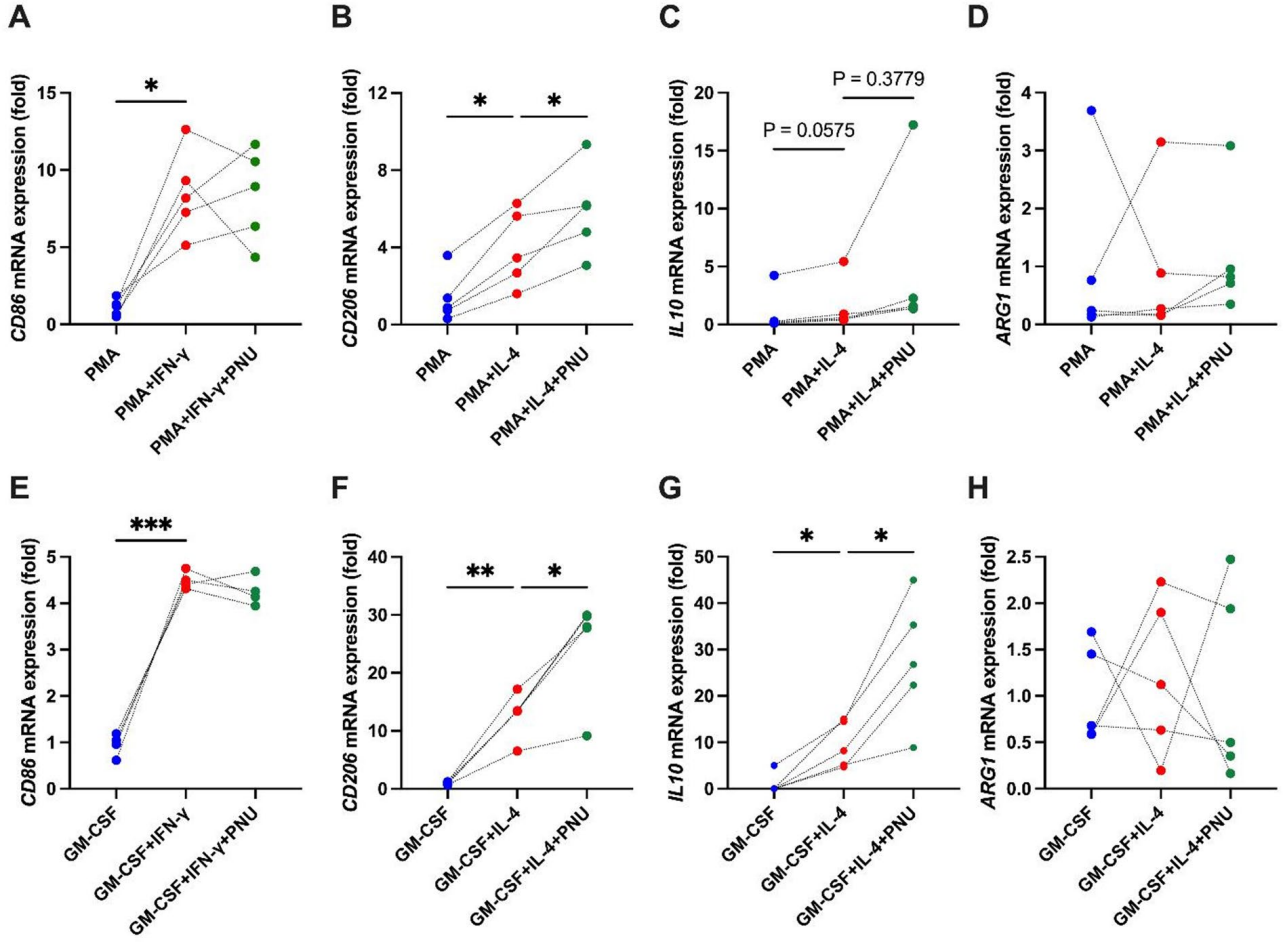
Effect of an α7nAChR agonist on the differentiation of THP-1 cells into M1/M2 macrophages. (**A**) mRNA expression of the M1 macrophage marker *CD86* after IFN-γ stimulation with or without PNU-282987 treatment in THP-1 cells (*N* = 5). (**B–D**) Expression of the M2 macrophage marker *CD206*, *IL10* and *ARG1* after IL-4 stimulation with or without PNU-282987 treatment in THP-1 cells (*N* = 5). (**E**) mRNA expression of the M1 macrophage marker *CD86* after IFN-γ stimulation with or without PNU-282987 treatment in human PBMC-derived macrophages (*N* = 5). (**F–H**) Expression of the M2 macrophage marker *CD206*, *IL10* and *ARG1* after IL-4 stimulation with or without PNU-282987 treatment in human PBMC-derived macrophages (*N* = 5). *p < 0.05, **p < 0.01, ***p < 0.001, denotes significant differences. The results of the same series are connected by a line. *CD86*, cluster of differentiation 86; *CD206*, cluster of differentiation 206; *IL10*, Interleukin-10; *ARG1*, arginase; PMA; Phorbol 12-myristate 13-acetate.

Finally, we verified whether α7nAChR activation can enhance the M2 phenotype in vitro. We hypothesized that α7nAChR activation affects M2 macrophage differentiation from monocytes, thereby promoting M2 dominance. We used the human monocytic leukemia cell line THP-1, which is commonly used in M1/M2 differentiation experiments^20^, to examine the expression of M1 and M2 macrophage markers during treatment with the α7nAChR agonist PNU-282987. THP-1 cells were stimulated with PMA to differentiate into macrophages and then stimulated with IFN-γ and IL-4 to differentiate into M1 and M2 macrophages, respectively. Treatment with PMA for 24 h significantly increased *CD68* mRNA expression (Supplementary Fig. S5A), thereby indicating the differentiation of monocytes into macrophages. Similarly, treatment with IFN-γ and IL-4 for 48 h significantly increased the mRNA expression levels of *CD86* and *CD206*, respectively, confirming the differentiation of monocytes into M1 and M2 macrophages (Fig. 6A, B). PNU-282987 treatment did not affect *CD86* mRNA expression but significantly enhanced *CD206* mRNA expression (Fig. 6A, B). Similarly, the expression of another M2 marker, *IL10*, was also increased by PNU-282987 (Fig. 6C). In contrast, although *NOS2* and *ARG1* were examined as additional M1/M2 markers, their expression did not increase in response to IFN-γ or IL-4 stimulation (Fig. 6D, Supplementary Fig. S5B). Therefore, in the present study, we considered these markers to be unsuitable for evaluating M1/M2 polarization.

Moreover, we also conducted experiments using hPBMC. When PNU-282987 was administered during M1 or M2 polarization by IFN-γ or IL-4, respectively, the expression of M1 markers such as *CD86* remained unchanged, whereas the expression of M2 markers including *CD206* was significantly increased by PNU-282987 (Fig. 6E, F). *IL10* was similarly upregulated by PNU-282987 (Fig. 6G), whereas *NOS2* and *ARG1*, as in THP-1 cells, did not show increased expression even after polarization stimuli (Fig. 6H, Supplementary Fig. S5C).

These results indicated that α7nAChR activation enhanced M2 macrophage differentiation.

## Discussion

**Fig. 7:**
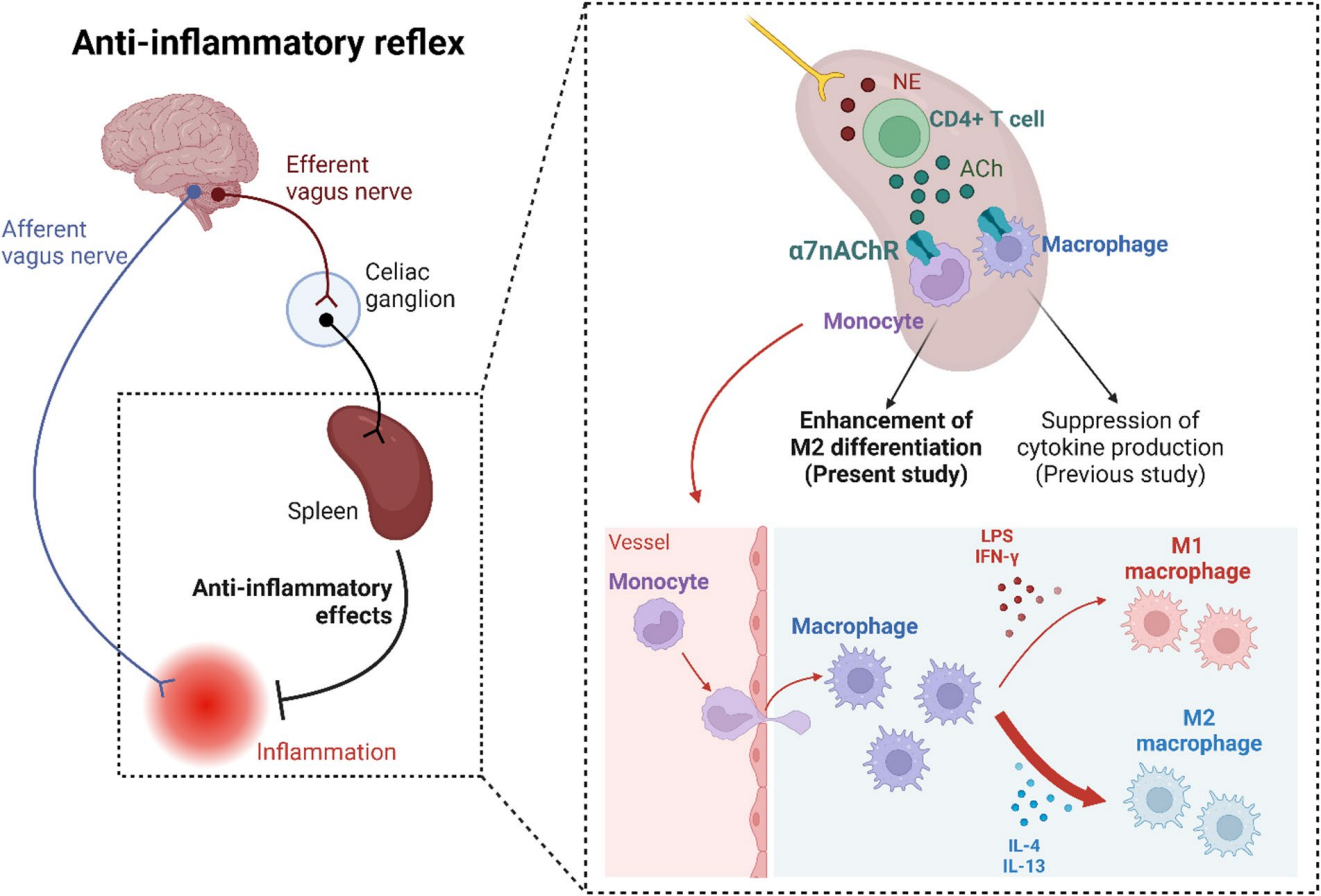
Enhanced M2 macrophage differentiation mediated by α7nAChR in the spleen. α7nAChR, α7 nicotinic acetylcholine receptor.

In the present study, we demonstrated that α7nAChR deficiency suppressed M2 dominance in vivo and that α7nAChR activation enhanced M2 macrophage differentiation from monocytes in vitro. Furthermore, we found that the α7nAChR-mediated differentiation of monocytes into M2 macrophages may be enhanced in the spleen. The previously established “anti-inflammatory reflex,” which involves α7nAChR, highlights the role of acetylcholine, produced by noradrenaline-stimulated CD4-positive T cells in the spleen, in stimulating α7nAChR on macrophages to suppress inflammation^7,8^. In the present study, we present novel findings showing that α7nAChR present on monocytes^18,19^ enhanced M2 differentiation in the spleen, suggesting that M2-differentiation-enhanced monocytes migrate to inflammatory sites to exert anti-inflammatory effects. This discovery expands our understanding of the “anti-inflammatory reflex” pathway mediated by α7nAChR (Fig. 7).

Presently, novel strategies are being developed to control inflammation by regulating neural pathways. For example, the implantation of a device capable of electrically stimulating the vagus nerve induced symptomatic relief in patients with refractory rheumatoid arthritis in a clinical trial by decreasing serum TNF-α, IL-1β, and IL-6 levels and reducing pathological scores^21^. Additionally, inflammatory bowel disease is suppressed by vagus nerve stimulation^22^. In both cases, the relief of symptoms has been attributed to the “anti-inflammatory reflex” via α7nAChR activation, which inhibits the release of inflammatory cytokines. Considering that the activation of α7nAChR can enhance M2 polarity, the therapeutic effect of the “anti-inflammatory reflex” via α7nAChR may not be due to the suppression of inflammatory cytokine secretion alone but also due to M2 polarity enhancement and the increased anti-inflammatory effects of M2 macrophages. These findings provide valuable insights into the mechanism of the “anti-inflammatory reflex” and its practical application. Moreover, it is plausible that α7nAChR-mediated modulation of macrophage polarity may influence disease severity and survival under acute inflammatory conditions. Future studies employing severe inflammation models will be required to determine whether α7nAChR-dependent enhancement of M2 polarization translates into improved systemic outcomes, including survival.

Epidemiological observation demonstrated that smoking exerts a protective effect against ulcerative colitis, attributed in part to the capacity of nicotine to enhance M2 macrophage polarization^23,24^. Although this represents a noteworthy epidemiological finding, the underlying mechanisms have remained unclear. Our study provides scientific evidence supporting this epidemiological observation by demonstrating that α7nAChR activation via nicotine intake through smoking might enhance monocyte differentiation into M2 macrophages and exert anti-inflammatory effects, thereby partially elucidating the mechanism for the protective effect of smoking in ulcerative colitis.

*Nos2* was upregulated in infectious inflammation, while its expression was almost absent in non-infectious inflammation. In infectious inflammation, pathogen-associated molecular patterns (PAMPs) stimulate pattern recognition receptors (PRRs) to trigger immune responses. In contrast, damage-associated molecular patterns (DAMPs) released by physical stimuli stimulate PRRs during non-infectious inflammation^25^. Therefore, it is possible that the downstream signals of PAMPs and DAMPs are different, resulting in differences in *Nos2* expression during infectious and non-infectious inflammation (Supplementary Fig. S6). The marked increase in *Nos2* expression in the early stages of infectious inflammation may have certain physiological implications. For instance, inducible nitric oxide synthase, encoded by *Nos2*, produces NO, which plays an important role in the defense against infection. NO reacts with reactive oxygen species and is converted to reactive nitrogen oxide species, such as peroxynitrite, which exhibit strong antimicrobial activity^26,27^. Therefore, the rapid induction of *Nos2* expression may be prioritized in infectious inflammation to rapidly eliminate pathogens.

This study has two main limitations. First, we did not differentiate between specific subtypes of M2 macrophages in vitro, namely, M2a, M2b, M2c, and M2d, all of which produce the anti-inflammatory cytokine IL-10 but exhibit distinct functions^28^. While our findings indicate that α7nAChR maintains the overall proportion of M2 macrophages, it remains unclear which specific subtype is most affected. Further classification of these subtypes would provide more detailed insights into the influence of α7nAChR on macrophage polarization. Second, our study focused on peritoneal inflammation due to the convenience of macrophage isolation, thus limiting the generalizability of our findings to inflammatory processes in other tissues, such as pulmonary inflammation, hepatitis, or intestinal inflammation. Expanding this research to include α7nAChR-deficient mouse models of these inflammation types could allow for a broader understanding of the systemic relevance of α7nAChR in modulating M2 macrophage proportions across different inflammatory environments.

Recently, therapeutic strategies for treating inflammatory diseases by regulating M1/M2 macrophage polarity have attracted considerable attention^29^; thus, the findings of the present study provide important insights for drug development.

## Supplementary Information

Below is the link to the electronic supplementary material.

Supplementary Material 1

Supplementary Material 2

## Data Availability

The datasets generated during and/or analyzed during the current study are available from the corresponding author upon reasonable request.
