# Supplementary material for: The role of the α7 nicotinic acetylcholine receptor in promoting M2 macrophage polarization at inflammatory sites
Source: Sci Rep. 2026 Jan 14;16:5267. doi: 10.1038/s41598-026-35757-2 (PMC12880967; doi:10.1038/s41598-026-35757-2)
Supplement: Supplementary file 1 — Supplementary Material 1 [file 41598_2026_35757_MOESM1_ESM.docx]

**The Role of the α7 Nicotinic Acetylcholine Receptor in Promoting M2 Macrophage Polarization at Inflammatory Sites**

Taiki Mihara^1,^*, Hiroshi Tanabe^1^, Yuma Nonoshita^1^, Yuki Yamakawa^1^, Tamaki Kurosawa^1^ and Masatoshi Hori^1^

^1^Department of Veterinary Pharmacology, Graduate School of Agriculture and Life Sciences, The University of Tokyo, Bunkyo-ku, Tokyo 113-8657, Japan

*Correspondence: Taiki Mihara (ORCID: 0009-0003-1611-8329)

[amihara@g.ecc.u-tokyo.ac.jp](mailto:amihara@g.ecc.u-tokyo.ac.jp)

Department of Veterinary Pharmacology, Graduate School of Agriculture and Life Sciences, The University of Tokyo, Bunkyo-ku, Tokyo 113-8657, Japan

Phone: +81-3-5841-5417

Fax: +81-3-5841-8183

**
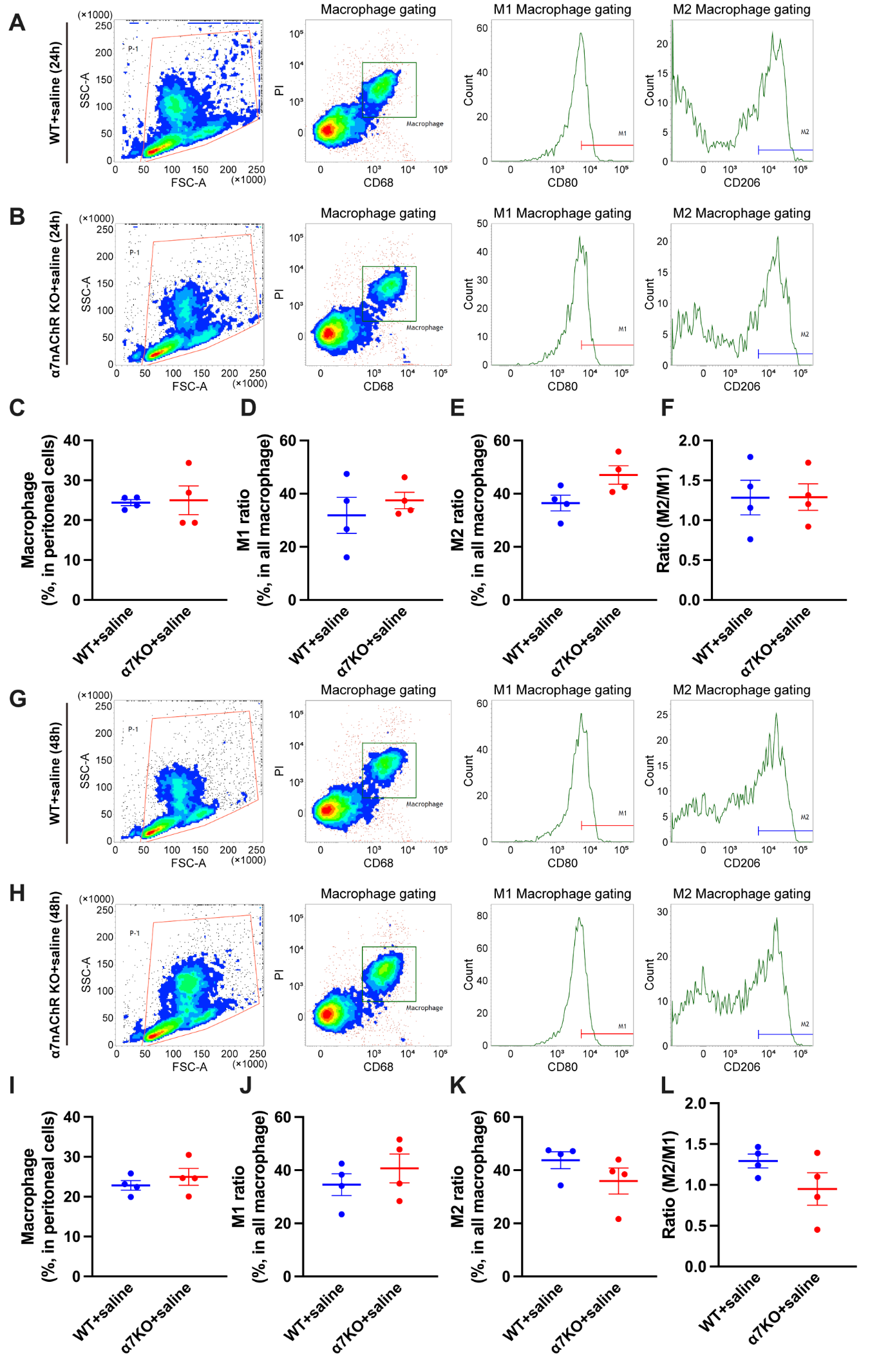
**

**Supplemental Figure 1**

Saline administration did not influence macrophage polarization in M1/M2 phenotype in peritoneal cell populations

(**A**) FACS plots of peritoneal cell populations derived from WT and α7KO mice 24 h after saline administration. The proportions of (**B**) macrophages to whole peritoneal cell population, (**C**) M1 macrophages to all macrophages, and (**D**) M2 macrophages to all macrophages are shown (N = 4). (**E**) Ratio of M2/M1 derived in (C) and (D) (N = 4). (**F**) FACS plots of peritoneal cell populations derived from WT and α7KO mice 48 h after saline administration. The proportions of (**G**) macrophages to whole peritoneal cell population, (**H**) M1 macrophages to all macrophages, and (**I**) M2 macrophages to all macrophages are shown (N = 4). (**J**) Ratio of M2/M1 derived in (H) and (I) (N = 4). WT, wild-type; α7KO, α7 nicotinic acetylcholine receptor knockout.


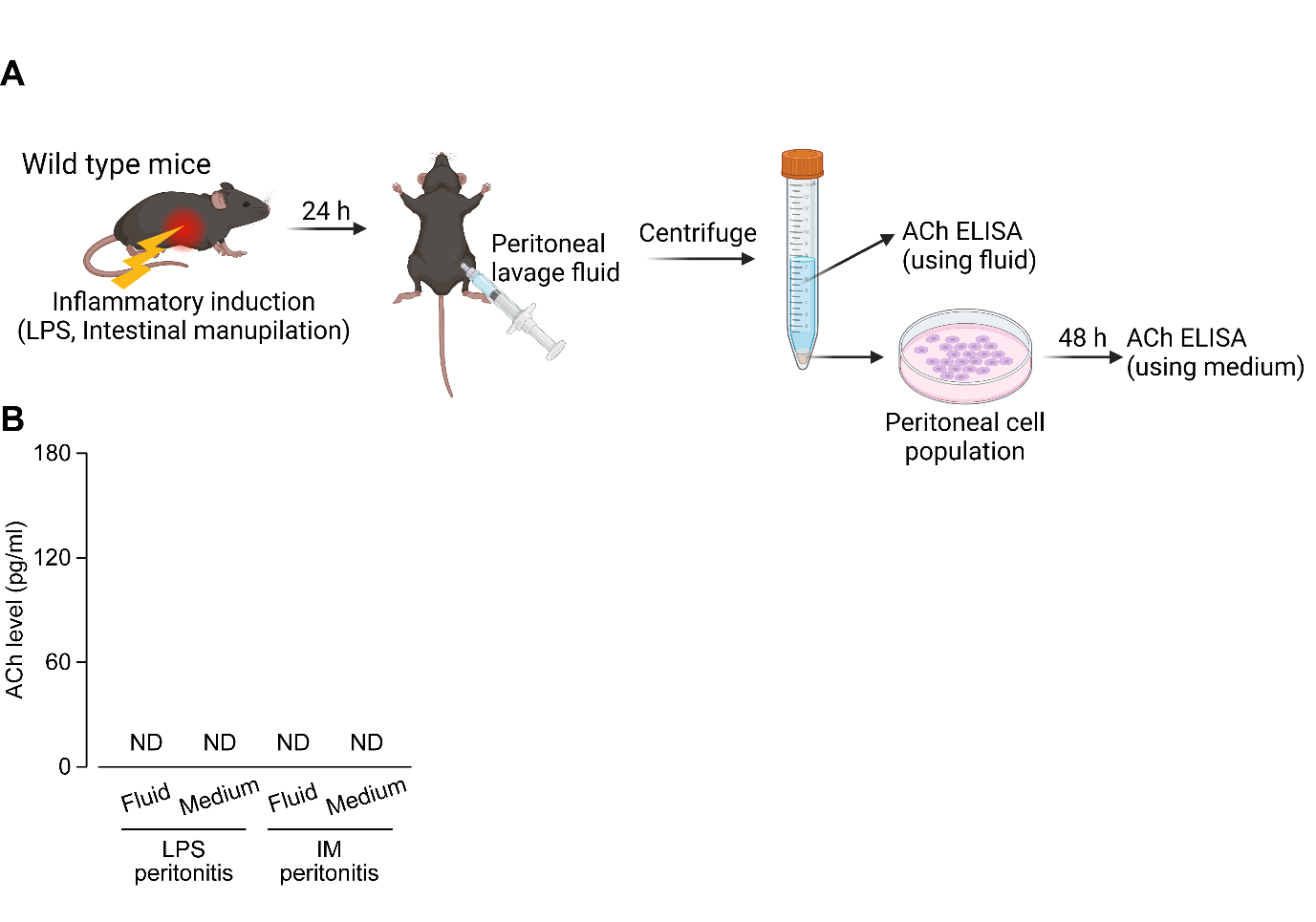
 **Supplemental Figure 2**

Acetylcholine was undetectable in peritoneal lavage fluids and peritoneal cell culture medium from LPS- and IM-induced peritonitis model mice.

IM, intestinal manipulation; ND, not detected


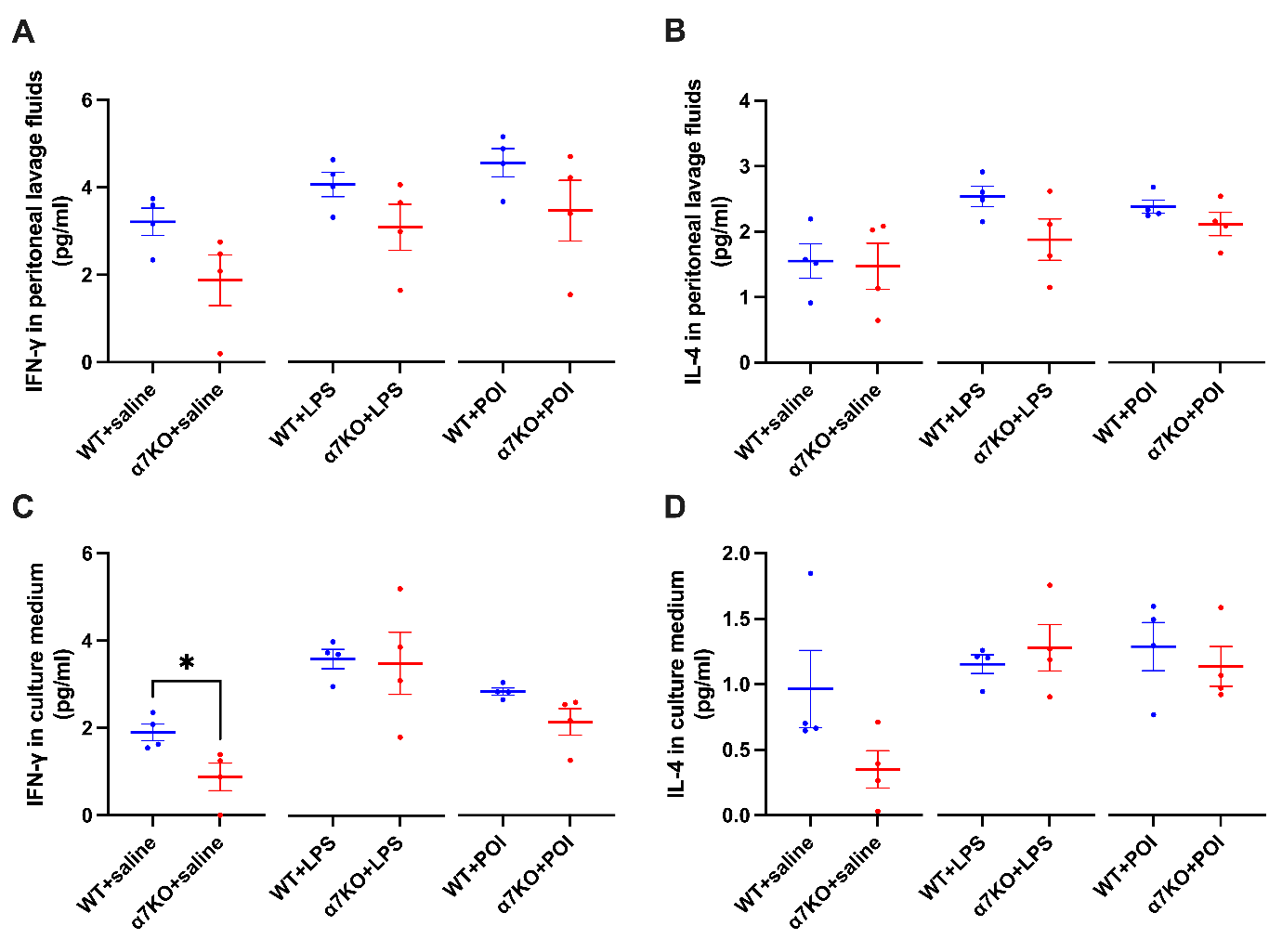
**Supplemental Figure 3**

(**A, B**) Levels of IFN-γ and IL-4 in peritoneal lavage fluids, (**C, D**) Levels of IFN-γ and IL-4 in culture medium of peritoneal cell populations obtained from LPS- and IM-induced peritonitis model mice.

**
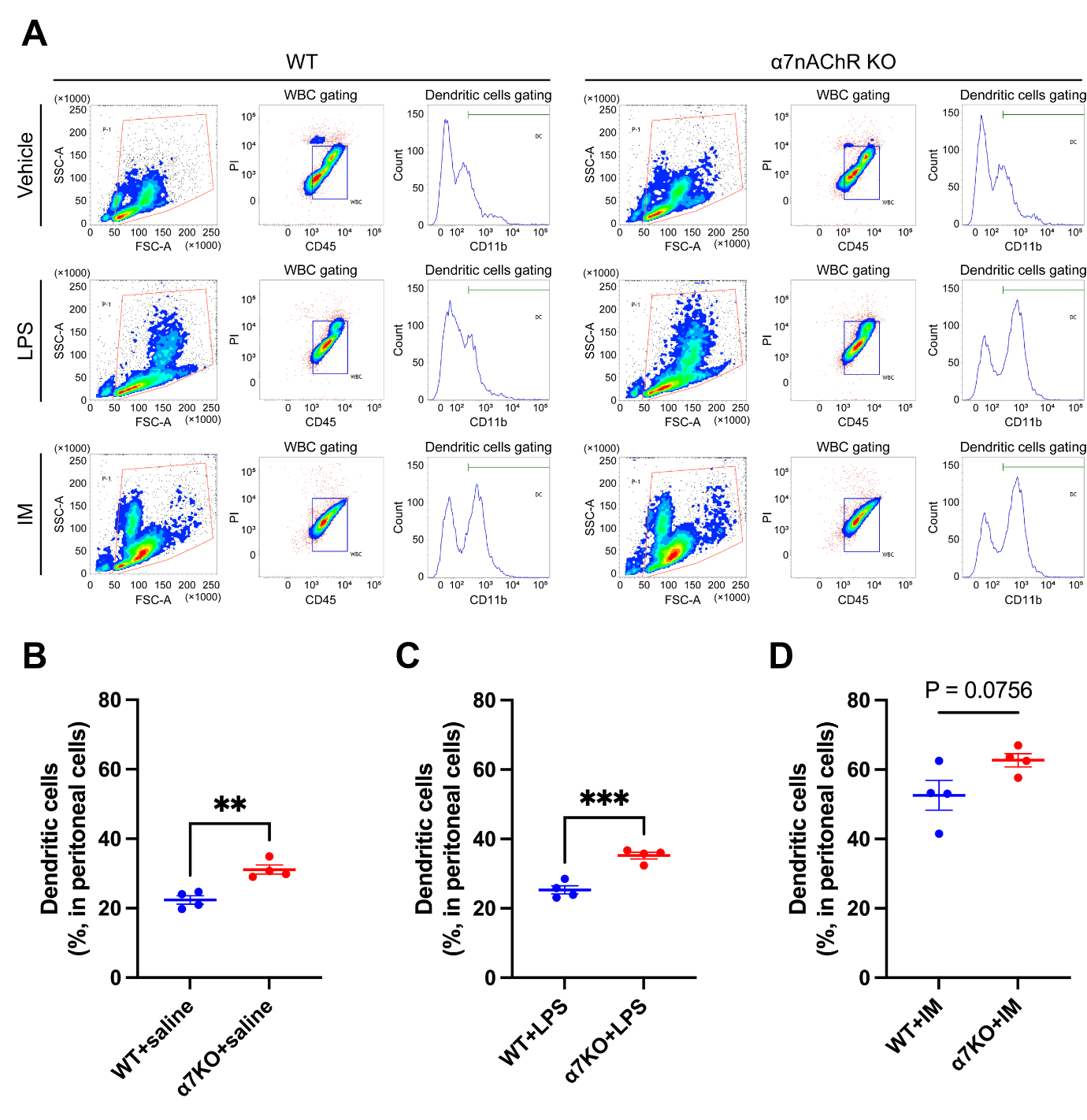
Supplemental Figure 4**

(**A**) Representative FACS plots of peritoneal cell populations from WT and α7nAChR-deficient (α7KO) mice 48 h after saline and LPS administration, or IM procedure. (**B-D**) The proportions of dendritic cells within the whole peritoneal cell population (N = 4).


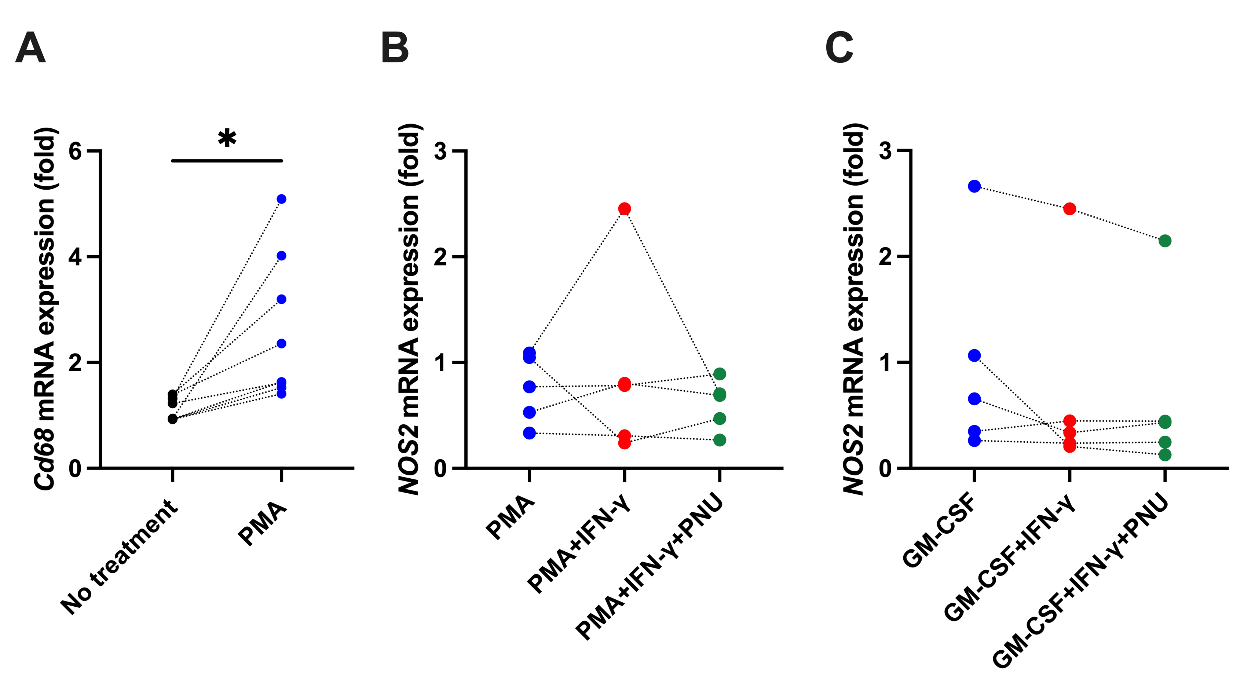
**Supplemental Figure 5**

(**A**) mRNA expression of the macrophage marker *CD68* in THP-1 cells treated with or without PMA (N = 8). (**B**) mRNA expression of the M1 macrophage marker *NOS2* in THP-1 cells after IFN-γ stimulation with or without PNU-282987 treatment (N = 5). (**C**) mRNA expression of the M1 macrophage marker *NOS2* in human PBMCs-derived macrophages after IFN-γ stimulation with or without PNU-282987 treatment (N = 5).


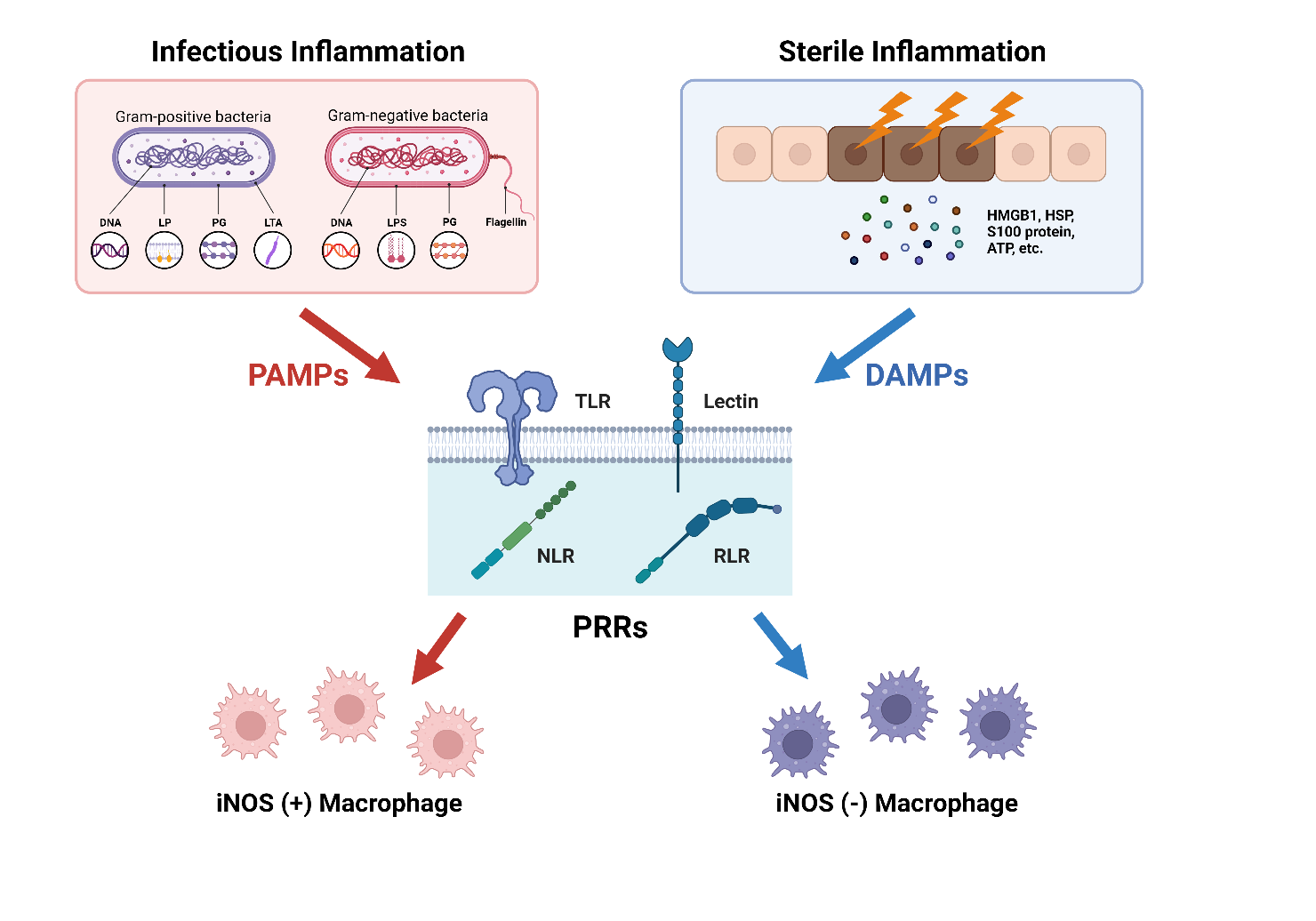
**Supplemental Figure 6**

Different subsets of macrophages induced in infectious and non-infectious inflammation.
