# Supplementary material for: The role of the α7 nicotinic acetylcholine receptor in promoting M2 macrophage polarization at inflammatory sites
Source: Sci Rep. 2026 Jan 14;16:5267. doi: 10.1038/s41598-026-35757-2 (PMC12880967; doi:10.1038/s41598-026-35757-2)
Supplement: Supplementary file 2 — Supplementary Material 2 [file 41598_2026_35757_MOESM2_ESM.docx]

**The Role of the α7 Nicotinic Acetylcholine Receptor in Promoting M2 Macrophage Polarization at Inflammatory Sites**

Taiki Mihara^1,^*, Hiroshi Tanabe^1^, Yuma Nonoshita^1^, Yuki Yamakawa^1^, Tamaki Kurosawa^1^ and Masatoshi Hori^1^

^1^Department of Veterinary Pharmacology, Graduate School of Agriculture and Life Sciences, The University of Tokyo, Bunkyo-ku, Tokyo 113-8657, Japan

*Correspondence: Taiki Mihara (ORCID: 0009-0003-1611-8329)

[amihara@g.ecc.u-tokyo.ac.jp](mailto:amihara@g.ecc.u-tokyo.ac.jp)

Department of Veterinary Pharmacology, Graduate School of Agriculture and Life Sciences, The University of Tokyo, Bunkyo-ku, Tokyo 113-8657, Japan

Phone: +81-3-5841-5417

Fax: +81-3-5841-8183

**Supplemental Table Ⅰ**

Specifics of the animal diet composition

| **Composition** | **Weight (g, in 100 g)** |
| --- | --- |
| Water | 8.1 |
| Crude protein | 23.2 |
| Crude fat | 4.9 |
| Ash | 5.9 |
| Crude fiber | 3.3 |
| NFE | 54.7 |

**Supplemental Table Ⅱ**

Primer sets used in this study.

| **Species** | **Gene** | **Expected size (bp)** | **Forward / Reverse** | **Sequence 5’→3’** |
| --- | --- | --- | --- | --- |
| Mouse | *Arg1* | 121 | Forward | TACATTGGCTTGCGAGACGTAGA |
|  |  |  | Reverse | AGGTCTCTTCCATCACCTTGCC |
|  | *Chat* | 165 | Forward | TGGATGAAACATACCTGATGAGCAA |
|  |  |  | Reverse | CGTGAAAGCTGGAGATGCAGAA |
|  | *Ccl1* | 144 | Forward | TGCTTACGGTCTCCAATAGCT |
|  |  |  | Reverse | AGCTTTCTCTACCTTTGTTCAGC |
|  | *Cd163* | 205 | Forward | CATCAGTGCCATTGGTCGAG |
|  |  |  | Reverse | ACTGCCTCCACCTACAAGTC |
|  | *Nos2* | 118 | Forward | ATTTTGCATGACACTCTTCACCAC |
|  |  |  | Reverse | TAGGCTTGTCTCTGGGTCCTCT |
|  | *RNA18S5* | 155 | Forward | AAACGGCTACCACATCCAAG |
|  |  |  | Reverse | CCTCCAATGGATCCTCGTTA |
|  | *Tnf* | 125 | Forward | CAAACCACCAAGTGGAGGAG |
|  |  |  | Reverse | GTAGACAAGGTACAACCCATCG |
|  | *Vegfa* | 141 | Forward | ACTGGACCCTGGCTTTACTG |
|  |  |  | Reverse | TCTCAATCGGACGGCAGTAG |
| Human | *ARG1* | 138 | Forward | ACTCCCTGTATATCTGCCAAGG |
|  |  |  | Reverse | CACCTTGCCAATTCCTAGTCTG |
|  | *CD68* | 202 | Forward | TCAGCTTTGGATTCATGCAG |
|  |  |  | Reverse | AGGTGGACAGCTGGTGAAAG |
|  | *CD86* | 160 | Forward | TGTACGACGTTTCCATCAGC |
|  |  |  | Reverse | ATCCAAGGAATGTGGTCTGG |
|  | *CD206* | 179 | Forward | ACGGACTGGGTTGCTATCAC |
|  |  |  | Reverse | TTCCACCTGCTCCATAAACC |
|  | *IL10* | 121 | Forward | ATCAAGGCGCATGTGAACTC |
|  |  |  | Reverse | CATTCTTCACCTGCTCCACG |
|  | *NOS2* | 180 | Forward | TCCAAATCTTGCCTGGGGTC |
|  |  |  | Reverse | CTTTGTTACCGCTTCCACCC |
|  | *RNA18S5* | 155 | Forward | CGTTCTTAGTTGGTGGAGCG |
|  |  |  | Reverse | AACGCCACTTGTCCCTCTAA |
